# Supplementary material for: A Likelihood Approach for Real-Time Calibration of Stochastic Compartmental Epidemic Models
Source: PLoS Comput Biol. 2017 Jan 17;13(1):e1005257. doi: 10.1371/journal.pcbi.1005257 (PMC5240920; doi:10.1371/journal.pcbi.1005257)
Supplement: S1 File — (TAR.GZ) [file pcbi.1005257.s014.tar.gz › HSPH_Online-SI-Revision/PF08/n1-extreme/n1-extreme_PF2_table-4.pdf]

1	newly 3 weeks spec	116.	49.02	{3., 105.}	60.2241	0.671131	newly 3 weeks cumul	225.	143.	{14., 305.}	55.2711	0.502538	newly 5 weeks spec	137.	41.36	{3., 87.}	69.8102	0.779441	newly 5 weeks cumul	508.	230.31	{24., 461.}	56.0531	0.574541	newly 64 weeks cumul	896.	463.83	{60., 794.}	48.2824	0.461891
2	newly 3 weeks spec	95.	36.19	{3., 72.}	62.2	0.656268	newly 3 weeks cumul	271.	112.8	{17., 207.}	59.2325	0.565314	newly 5 weeks spec	80.	32.92	{3., 73.}	64.1	0.686268	newly 5 weeks cumul	464.	181.18	{22., 349.}	61.1207	0.594457	newly 60 weeks cumul	752.	351.01	{39., 671.}	54.5013	0.524395
3	newly 3 weeks spec	74.	41.22	{8., 82.}	50.5946	0.512632	newly 3 weeks cumul	153.	123.68	{23., 249.}	50.3791	0.404021	newly 5 weeks spec	91.	35.43	{3., 69.}	63.4615	0.685119	newly 5 weeks cumul	361.	197.8	{29., 375.}	48.687	0.48838	newly 62 weeks cumul	847.	401.25	{68., 722.}	53.4109	0.541253
4	newly 3 weeks spec	66.	37.87	{1., 69.}	50.0455	0.589454	newly 3 weeks cumul	162.	116.27	{5., 226.}	48.9444	0.478835	newly 5 weeks spec	58.	30.23	{0., 60.}	53.3276	0.616711	newly 5 weeks cumul	300.	181.61	{8., 338.}	47.53	0.501519	newly 61 weeks cumul	732.	388.66	{60., 679.}	48.0792	0.469746
5	newly 3 weeks spec	89.	25.78	{3., 49.}	71.0337	0.769914	newly 3 weeks cumul	241.	83.35	{14., 152.}	65.7801	0.615367	newly 5 weeks spec	98.	20.47	{0., 43.}	79.1122	0.975559	newly 5 weeks cumul	447.	126.6	{18., 236.}	71.6779	0.721549	newly 54 weeks cumul	764.	253.63	{18., 534.}	66.8024	0.701537
6	newly 3 weeks spec	46.	30.45	{2., 63.}	53.3696	0.547377	newly 3 weeks cumul	105.	100.46	{5., 201.}	62.1714	0.443888	newly 5 weeks spec	44.	23.26	{0., 50.}	56.6364	0.67837	newly 5 weeks cumul	246.	150.61	{10., 310.}	51.2073	0.498357	newly 59 weeks cumul	557.	292.82	{21., 547.}	51.6338	0.531983
7	newly 3 weeks spec	75.	45.45	{5., 87.}	49.6667	0.490596	newly 3 weeks cumul	185.	140.86	{18., 276.}	51.8919	0.425868	newly 5 weeks spec	81.	37.68	{6., 76.}	55.4074	0.55259	newly 5 weeks cumul	355.	219.57	{32., 409.}	47.6873	0.431327	newly 63 weeks cumul	753.	440.74	{109., 781.}	43.4343	0.384172
8	newly 3 weeks spec	76.	36.8	{11., 70.}	55.7105	0.46437	newly 3 weeks cumul	224.	116.77	{40., 211.}	53.0045	0.385978	newly 5 weeks spec	72.	31.7	{6., 63.}	56.4167	0.532061	newly 5 weeks cumul	382.	182.82	{55., 335.}	54.4817	0.418102	newly 57 weeks cumul	642.	358.76	{102., 640.}	46.4019	0.365638
9	newly 3 weeks spec	86.	52.45	{4., 105.}	53.0349	0.504793	newly 3 weeks cumul	199.	151.77	{14., 305.}	52.9497	0.427817	newly 5 weeks spec	96.	43.63	{4., 88.}	55.3021	0.575932	newly 5 weeks cumul	400.	244.05	{32., 461.}	48.1625	0.447441	newly 64 weeks cumul	731.	477.05	{85., 794.}	38.409	0.352814
10	newly 3 weeks spec	73.	39.37	{12., 75.}	54.0411	0.41441	newly 3 weeks cumul	217.	123.7	{45., 225.}	51.6313	0.361339	newly 5 weeks spec	62.	31.45	{10., 64.}	53.2742	0.431341	newly 5 weeks cumul	362.	190.31	{71., 341.}	52.5994	0.383936	newly 57 weeks cumul	751.	393.07	{142., 653.}	48.1718	0.35547
11	newly 3 weeks spec	88.	44.95	{3., 88.}	53.8977	0.600692	newly 3 weeks cumul	241.	142.75	{18., 286.}	52.4108	0.483163	newly 5 weeks spec	81.	36.42	{4., 76.}	57.2346	0.608621	newly 5 weeks cumul	416.	219.62	{27., 414.}	51.1923	0.491289	newly 63 weeks cumul	680.	433.25	{105., 778.}	41.1868	0.369669
12	newly 3 weeks spec	65.	46.77	{2., 104.}	59.6154	0.562073	newly 3 weeks cumul	159.	134.87	{14., 290.}	62.6981	0.465323	newly 5 weeks spec	109.	39.89	{3., 87.}	63.4587	0.704188	newly 5 weeks cumul	387.	218.95	{21., 453.}	52.9276	0.514085	newly 64 weeks cumul	698.	458.98	{60., 803.}	40.063	0.377916
13	newly 3 weeks spec	49.	34.16	{12., 64.}	44.7347	0.294909	newly 3 weeks cumul	125.	113.77	{44., 189.}	43.112	0.217907	newly 5 weeks spec	41.	27.41	{8., 54.}	49.0976	0.34899	newly 5 weeks cumul	222.	172.02	{64., 318.}	43.5045	0.253399	newly 57 weeks cumul	659.	345.74	{124., 628.}	49.6753	0.374678
14	newly 3 weeks spec	113.	39.17	{5., 71.}	65.3363	0.673789	newly 3 weeks cumul	293.	122.2	{24., 224.}	58.2935	0.537481	newly 5 weeks spec	92.	33.9	{3., 67.}	66.3696	0.69799	newly 5 weeks cumul	501.	194.49	{32., 345.}	61.1796	0.574231	newly 61 weeks cumul	659.	378.19	{67., 711.}	47.3551	0.428326
15	newly 3 weeks spec	61.	30.24	{10., 62.}	54.3934	0.433744	newly 3 weeks cumul	196.	98.84	{38., 180.}	52.449	0.380536	newly 5 weeks spec	65.	22.77	{6., 50.}	65.6462	0.642625	newly 5 weeks cumul	338.	149.09	{52., 287.}	57.5	0.448107	newly 56 weeks cumul	615.	285.15	{69., 535.}	55.0293	0.460202
16	newly 3 weeks spec	79.	40.73	{1., 88.}	56.3418	0.641985	newly 3 weeks cumul	198.	124.28	{15., 268.}	54.7778	0.489121	newly 5 weeks spec	51.	34.91	{2., 74.}	53.1961	0.54752	newly 5 weeks cumul	335.	197.51	{27., 406.}	51.2925	0.486663	newly 63 weeks cumul	677.	412.61	{58., 699.}	42.9527	0.418409
17	newly 3 weeks spec	73.	28.24	{1., 66.}	62.274	0.745282	newly 3 weeks cumul	179.	92.03	{5., 183.}	53.9832	0.5554	newly 5 weeks spec	61.	24.5	{0., 57.}	62.5574	0.770495	newly 5 weeks cumul	321.	142.94	{15., 292.}	57.6199	0.60581	newly 60 weeks cumul	748.	308.51	{33., 600.}	59.2634	0.620281
18	newly 3 weeks spec	100.	42.07	{4., 90.}	60.07	0.689689	newly 3 weeks cumul	211.	134.21	{17., 268.}	52.109	0.470764	newly 5 weeks spec	103.	36.36	{2., 77.}	64.699	0.779104	newly 5 weeks cumul	426.	210.03	{27., 406.}	53.1854	0.526051	newly 63 weeks cumul	855.	433.73	{58., 752.}	49.4865	0.466244
19	newly 3 weeks spec	67.	49.48	{2., 105.}	55.2537	0.544571	newly 3 weeks cumul	165.	142.96	{13., 285.}	58.7394	0.476297	newly 5 weeks spec	77.	41.06	{1., 85.}	52.8052	0.621527	newly 5 weeks cumul	334.	229.84	{17., 435.}	49.8443	0.484397	newly 64 weeks cumul	820.	468.02	{56., 781.}	43.1512	0.456142
20	newly 3 weeks spec	98.	35.82	{11., 70.}	64.0204	0.540931	newly 3 weeks cumul	274.	118.12	{45., 223.}	58.0146	0.444598	newly 5 weeks spec	84.	26.31	{5., 53.}	68.7262	0.694019	newly 5 weeks cumul	458.	175.52	{65., 321.}	61.8166	0.498642	newly 57 weeks cumul	701.	321.16	{91., 584.}	54.311	0.434668
21	newly 3 weeks spec	44.	28.88	{2., 66.}	56.5909	0.538978	newly 3 weeks cumul	115.	93.49	{9., 197.}	58.113	0.440032	newly 5 weeks spec	43.	22.93	{1., 53.}	58.3023	0.623297	newly 5 weeks cumul	219.	142.55	{16., 292.}	53.9772	0.468543	newly 60 weeks cumul	654.	318.63	{38., 606.}	54.026	0.513156
22	newly 3 weeks spec	79.	37.19	{3., 72.}	55.6582	0.588401	newly 3 weeks cumul	241.	122.34	{16., 220.}	52.3402	0.495621	newly 5 weeks spec	63.	29.64	{2., 57.}	59.4286	0.644039	newly 5 weeks cumul	388.	185.39	{17., 347.}	53.5851	0.518432	newly 61 weeks cumul	683.	341.4	{59., 622.}	52.3514	0.495913
23	newly 3 weeks spec	72.	41.11	{4., 90.}	55.3472	0.587266	newly 3 weeks cumul	168.	128.76	{16., 263.}	56.9048	0.453459	newly 5 weeks spec	79.	36.53	{2., 77.}	56.1139	0.669672	newly 5 weeks cumul	346.	204.66	{27., 408.}	50.815	0.481858	newly 63 weeks cumul	679.	432.89	{58., 752.}	40.7703	0.382582
24	newly 3 weeks spec	106.	51.07	{7., 105.}	58.4434	0.619657	newly 3 weeks cumul	236.	143.55	{14., 285.}	52.7585	0.500273	newly 5 weeks spec	137.	43.02	{3., 88.}	68.5985	0.759521	newly 5 weeks cumul	531.	233.94	{24., 435.}	57.4915	0.578383	newly 64 weeks cumul	842.	486.32	{85., 794.}	42.4347	0.3998
25	newly 3 weeks spec	63.	36.63	{2., 73.}	52.0476	0.567903	newly 3 weeks cumul	139.	113.4	{5., 239.}	55.3957	0.469037	newly 5 weeks spec	68.	30.34	{0., 62.}	59.2059	0.687142	newly 5 weeks cumul	276.	178.2	{8., 347.}	49.4275	0.49302	newly 61 weeks cumul	696.	377.09	{45., 679.}	48.3779	0.485106
26	newly 3 weeks spec	131.	43.81	{3., 87.}	67.4122	0.758272	newly 3 weeks cumul	280.	136.5	{11., 263.}	54.6786	0.553741	newly 5 weeks spec	153.	37.59	{2., 76.}	75.4314	0.901309	newly 5 weeks cumul	589.	216.76	{19., 410.}	63.4126	0.6583	newly 63 weeks cumul	887.	437.36	{74., 743.}	50.805	0.483487
27	newly 3 weeks spec	61.	37.56	{13., 71.}	47.0492	0.315154	newly 3 weeks cumul	160.	116.28	{46., 200.}	40.4625	0.245881	newly 5 weeks spec	54.	30.51	{11., 54.}	47.7222	0.362271	newly 5 weeks cumul	277.	180.65	{74., 316.}	43.3321	0.274679	newly 58 weeks cumul	659.	393.7	{180., 638.}	42.8164	0.291944
28	newly 3 weeks spec	77.	40.78	{3., 77.}	53.8182	0.558986	newly 3 weeks cumul	250.	128.68	{14., 243.}	53.088	0.524635	newly 5 weeks spec	99.	31.35	{2., 61.}	68.3737	0.777843	newly 5 weeks cumul	460.	197.06	{22., 371.}	58.1	0.586174	newly 62 weeks cumul	803.	386.57	{68., 660.}	52.2055	0.497192
29	newly 3 weeks spec	145.	37.52	{13., 75.}	74.1241	0.672213	newly 3 weeks cumul	304.	112.55	{46., 210.}	63.0033	0.498154	newly 5 weeks spec	113.	32.36	{9., 61.}	71.3628	0.679702	newly 5 weeks cumul	568.	180.42	{68., 322.}	68.2359	0.562922	newly 59 weeks cumul	875.	421.42	{140., 673.}	51.9086	0.382972
30	newly 3 weeks spec	52.	26.97	{5., 56.}	53.75	0.463566	newly 3 weeks cumul	145.	95.5	{27., 190.}	47.2552	0.326333	newly 5 weeks spec	41.	21.07	{2., 43.}	54.8537	0.531048	newly 5 weeks cumul	245.	140.16	{36., 275.}	49.1755	0.373635	newly 54 weeks cumul	398.	262.22	{57., 493.}	47.3568	0.365792
31	newly 3 weeks spec	115.	44.39	{4., 91.}	62.6696	0.69818	newly 3 weeks cumul	258.	140.04	{15., 272.}	52.1705	0.509316	newly 5 weeks spec	94.	38.54	{4., 78.}	59.3617	0.663597	newly 5 weeks cumul	471.	220.31	{28., 409.}	54.9575	0.539361	newly 63 weeks cumul	796.	452.83	{87., 758.}	43.8078	0.397435
32	newly 3 weeks spec	106.	42.71	{4., 90.}	61.2925	0.70026	newly 3 weeks cumul	229.	133.67	{15., 266.}	51.8821	0.500872	newly 5 weeks spec	128.	37.11	{4., 77.}	71.0078	0.833182	newly 5 weeks cumul	487.	210.51	{27., 408.}	58.0021	0.584571	newly 63 weeks cumul	773.	438.45	{82., 752.}	44.3946	0.42039
33	newly 3 weeks spec	60.	43.9	{15., 91.}	48.1	0.331318	newly 3 weeks cumul	152.	134.92	{53., 252.}	44.75	0.238943	newly 5 weeks spec	78.	35.95	{11., 68.}	56.2692	0.468121	newly 5 weeks cumul	313.	209.39	{81., 402.}	44.0351	0.287168	newly 59 weeks cumul	799.	436.27	{166., 711.}	46.174	0.328853
34	newly 3 weeks spec	85.	43.18	{3., 86.}	52.8471	0.598699	newly 3 weeks cumul	234.	129.34	{14., 246.}	48.8462	0.501621	newly 5 weeks spec	96.	36.33	{2., 72.}	62.9896	0.713203	newly 5 weeks cumul	427.	207.26	{23., 395.}	52.0562	0.546729	newly 62 weeks cumul	764.	415.39	{44., 736.}	47.25	0.488179
35	newly 3 weeks spec	73.	42.16	{4., 88.}	53.1507	0.56786	newly 3 weeks cumul	181.	127.57	{15., 266.}	53.7735	0.453702	newly 5 weeks spec	79.	36.15	{2., 75.}	55.8608	0.634772	newly 5 weeks cumul	361.	203.16	{28., 407.}	50.6316	0.486862	newly 63 weeks cumul	749.	422.85	{60., 743.}	45.2029	0.435885
36	newly 3 weeks spec	81.	33.27	{0., 72.}	59.5926	0.747758	newly 3 weeks cumul	195.	107.26	{5., 210.}	51.2718	0.540137	newly 5 weeks spec	83.	27.27	{0., 59.}	67.7711	0.852841	newly 5 weeks cumul	376.	165.28	{6., 334.}	57.2713	0.620942	newly 60 weeks cumul	755.	331.46	{26., 606.}	56.7576	0.587032
37	newly 3 weeks spec	78.	27.78	{7., 49.}	64.3846	0.620202	newly 3 weeks cumul	218.	92.99	{33., 168.}	58.1239	0.487751	newly 5 weeks spec	65.	21.91	{2., 40.}	66.2923	0.683009	newly 5 weeks cumul	380.	140.92	{46., 257.}	63.2	0.561084	newly 55 weeks cumul	728.	260.79	{49., 445.}	64.1772	0.606456
38	newly 3 weeks spec	93.	35.61	{2., 77.}	61.7097	0.676269	newly 3 weeks cumul	234.	115.33	{12., 228.}	53.8846	0.526421	newly 5 weeks spec	114.	29.89	{0., 63.}	74.1667	0.997483	newly 5 weeks cumul	462.	178.87	{16., 369.}	61.2835	0.636066	newly 60 weeks cumul	754.	339.17	{22., 645.}	55.6432	0.584474
39	newly 3 weeks spec	89.	43.5	{3., 87.}	55.6629	0.604693	newly 3 weeks cumul	179.	136.45	{11., 263.}	53.9274	0.449423	newly 5 weeks spec	117.	37.29	{2., 76.}	68.1282	0.787966	newly 5 weeks cumul	414.	216.38	{19., 410.}	51.0386	0.516967	newly 63 weeks cumul	855.	435.39	{74., 743.}	49.3275	0.46978
40	newly 3 weeks spec	70.	37.98	{5., 75.}	53.5714	0.548269	newly 3 weeks cumul	153.	125.59	{19., 230.}	47.6797	0.360613	newly 5 weeks spec	66.	27.71	{3., 52.}	60.5606	0.674284	newly 5 weeks cumul	299.	186.29	{34., 339.}	47.2876	0.420723	newly 61 weeks cumul	575.	318.05	{59., 570.}	48.3461	0.452534
41	newly 3 weeks spec	45.	32.82	{10., 59.}	47.7778	0.315994	newly 3 weeks cumul	135.	105.92	{42., 187.}	43.6593	0.257195	newly 5 weeks spec	55.	28.93	{9., 54.}	53.8727	0.437451	newly 5 weeks cumul	248.	165.91	{68., 292.}	45.246	0.298822	newly 56 weeks cumul	562.	353.89	{120., 577.}	41.6352	0.305256
42	newly 3 weeks spec	88.	50.45	{2., 105.}	54.6477	0.594925	newly 3 weeks cumul	235.	146.47	{13., 288.}	52.4809	0.519747	newly 5 weeks spec	105.	41.56	{1., 88.}	60.5905	0.725573	newly 5 weeks cumul	462.	234.24	{17., 435.}	52.671	0.559431	newly 64 weeks cumul	862.	463.95	{61., 783.}	46.2726	0.475655
43	newly 3 weeks spec	82.	40.87	{3., 87.}	55.061	0.602301	newly 3 weeks cumul	219.	128.52	{15., 259.}	53.4886	0.500182	newly 5 weeks spec	75.	36.97	{3., 76.}	53.4267	0.611049	newly 5 weeks cumul	390.	206.16	{25., 405.}	51.2923	0.510548	newly 63 weeks cumul	860.	437.42	{58., 763.}	49.3163	0.476146
44	newly 3 weeks spec	86.	36.73	{3., 69.}	58.5	0.622228	newly 3 weeks cumul	240.	112.37	{18., 218.}	54.6125	0.524622	newly 5 weeks spec	76.	32.67	{1., 67.}	62.6447	0.711157	newly 5 weeks cumul	408.	181.73	{18., 330.}	55.9926	0.552452	newly 61 weeks cumul	762.	369.06	{28., 711.}	52.8766	0.533913
45	newly 3 weeks spec	79.	49.35	{2., 104.}	54.6709	0.565608	newly 3 weeks cumul	203.	144.47	{13., 285.}	54.3695	0.491535	newly 5 weeks spec	105.	41.46	{1., 90.}	60.6857	0.70777	newly 5 weeks cumul	422.	231.71	{14., 453.}	50.9645	0.526658	newly 64 weeks cumul	886.	449.05	{61., 781.}	49.3239	0.488196
46	newly 3 weeks spec	93.	38.62	{2., 79.}	60.0215	0.678958	newly 3 weeks cumul	223.	118.18	{6., 239.}	52.5022	0.521898	newly 5 weeks spec	79.	33.62	{0., 72.}	61.2658	0.727883	newly 5 weeks cumul	409.	188.56	{16., 356.}	55.1834	0.569614	newly 61 weeks cumul	815.	393.92	{40., 724.}	52.454	0.53074
47	newly 3 weeks spec	63.	40.14	{3., 80.}	49.1429	0.522077	newly 3 weeks cumul	164.	122.35	{19., 245.}	50.7988	0.460564	newly 5 weeks spec	67.	34.55	{2., 69.}	57.0597	0.629583	newly 5 weeks cumul	308.	194.59	{25., 375.}	47.5617	0.496161	newly 62 weeks cumul	785.	394.28	{44., 722.}	51.4548	0.567453
48	newly 3 weeks spec	60.	42.69	{4., 84.}	47.7833	0.460198	newly 3 weeks cumul	141.	131.08	{19., 246.}	56.1702	0.405751	newly 5 weeks spec	68.	35.58	{2., 69.}	53.1471	0.605752	newly 5 weeks cumul	280.	207.13	{26., 371.}	45.2321	0.418822	newly 62 weeks cumul	672.	405.56	{44., 722.}	44.8214	0.452302
49	newly 3 weeks spec	96.	47.29	{3., 103.}	57.3229	0.597963	newly 3 weeks cumul	261.	136.84	{9., 278.}	54.0843	0.561638	newly 5 weeks spec	115.	39.47	{3., 84.}	65.6783	0.70837	newly 5 weeks cumul	493.	219.87	{17., 433.}	56.785	0.590798	newly 64 weeks cumul	853.	481.65	{85., 783.}	43.5885	0.426352
50	newly 3 weeks spec	57.	44.26	{5., 84.}	45.0175	0.402759	newly 3 weeks cumul	158.	144.18	{21., 251.}	47.2152	0.346379	newly 5 weeks spec	54.	34.97	{3., 68.}	48.5741	0.495204	newly 5 weeks cumul	272.	219.57	{27., 388.}	42.8934	0.353908	newly 62 weeks cumul	579.	406.57	{80., 697.}	39.6874	0.354895
